# MRI-based nomogram for differentiation of ovarian fibrothecoma and broad ligament myoma

**DOI:** 10.1038/s41598-022-12218-0

**Published:** 2022-05-17

**Authors:** Jingya Chen, Hailei Gu, Yu zhang, Weimin Fan, Shuai Chen, Yajing Wang, Ting Wu, Wenwei Tang, Zhongqiu Wang

**Affiliations:** 1 Department of Radiology, Affiliated Hospital of Nanjing University of Chinese Medicine, Nanjing, Jiangsu Province China; 2 Department of Radiology, Women’s Hospital of Nanjing Medical University (Nanjing Maternity and Child Health Care Hospital), Nanjing, Jiangsu Province China; 3 Department of Nuclear Medicine, Affiliated Hospital of Nanjing University of Chinese Medicine, Nanjing, Jiangsu Province China; 4 Department of Clinical Laboratory, Women’s Hospital of Nanjing Medical University (Nanjing Maternity and Child Health Care Hospital), Nanjing, Jiangsu Province China

**Keywords:** Medical research, Oncology

## Abstract

Currently, there are no effective approaches for differentiating ovarian fibrothecoma (OF) from broad ligament myoma (BLM). This retrospective study aimed to construct a nomogram prediction model based on MRI to differentiate OF from BLM. The quantitative and qualitative MRI features of 41 OFs and 51 BLMs were compared. Three models were established based on the combination of these features. The ability of the models to differentiate between the two cancers was assessed by ROC analysis. A nomogram based on the best model was constructed for clinical application. The three models showed good performance in differentiating between OF and BLM. The areas under the curve (AUC) of the models based on quantitative and qualitative variables were 0.88 (95% CI: 0.79–0.96) and 0.85 (95% CI: 0.76–0.93), respectively. The combined model designed from the significant variables exhibited the best diagnostic performance with the highest AUC of 0.92 (95% CI: 0.86–0.98). Calibration of the nomogram showed that the predicted probability matched the actual probability well. Analysis of the decision curve demonstrated that the nomogram was clinically useful. Relative T1 value, stone paving sign, enhancement patterns, and ascites were identified as valuable predictors for identifying OF or BLM. The MRI-based nomogram can serve as a preoperative tool to differentiate OF from BLM.

## Introduction

Ovarian fibrothecoma (OF) is a rare type of ovarian tumor that originates from the sex-cord stroma. There are several types of OFs, including fibroma, fibrothecoma, and thecoma, each with a different proportion of fibroblasts and theca cells^1^. Although most OFs are benign^2^, surgery is recommended for tumor removal. Ovarian fibrothecoma is the most common solid ovarian tumor^3^, with similar imaging features to those of uterine leiomyomas.

Uterine leiomyomas are the most common benign gynecologic tumors^4^. Notably, extrauterine leiomyomas may originate from the fallopian tubes of the uterus or broad ligament (5). Leiomyoma, particularly broad ligament myoma (BLM), is often misdiagnosed as OF preoperatively owing to its solid nature. It is also misdiagnosed as originating from the ovaries^5^. Pharmacotherapy or hormonal therapy is recommended for symptomatic patients.

Yin et al.^6^ found that combining conventional MRI and diffusion-weighted imaging (DWI) could effectively differentiate OF from malignant solid pelvic tumors. However, owing to the overlap of tumor components and imaging characteristics, it is challenging to distinguish OF from BLM preoperatively. Moreover, it is difficult to differentiate between OF and BLM based on preoperative quantitative and qualitative MRI features.

This retrospective study aimed to develop quantitative and qualitative MRI-based nomogram for preoperative differentiation between OF and BLMs.

## Material and methods

### Study population

This study protocol was performed in accordance with the guidelines outlined in the Declaration of Helsinki and was approved by the Ethics Committee of the Affiliated Hospital of Nanjing University of Chinese Medicine, and the requirement for written informed consent was waived due to the retrospective nature of the study.

A total of 53 patients with histologically proven OF and 97 patients with histologically confirmed extrauterine fibroids were enrolled from May 2016 to May 2020 and May 2017 to May 2020, respectively. Overall, 12 patients with OF and 57 patients with BLM were excluded based on the following criteria: (1) the preoperative MRI scan was absent or lacked one of the following sequences: DWI, apparent diffusion coefficient (ADC) map, or dynamic contrast-enhanced (DCE) (*n* = 24); (2) the tumor lesions were too small(< 1 cm)^7^to be measure on MRI (*n* = 3); (3) the myoma was not located in the broad ligament (*n* = 35); and (4) poor image quality to allow proper assessment (*n* = 7). Ultimately, 91 patients were enrolled in this study.

### MRI protocol

All MRI scans were performed using a 1.5-T MRI equipment (Magnetom Aera; Siemens Healthcare, Erlangen, Germany) with an abdominal phased-array coil. The MRI scanning parameters are listed in Table 1. Diffusion-weighted imaging (DWI) features were obtained with b-values of 0 and 1000 s/mm^2^. The ADC maps were automatically generated. Finally, DCE images were acquired after injection of contrast medium in the sagittal and axial planes. DCE scanning is performed after intravenous pumping administration of gadolinium (0.1 mmol/kg) using VIBE sequence. (pre-contrast in the axial and sagittal planes; post-contrast at 30 s, 60 s and 120 s in the sagittal and 180 s in the axial plane).

**Table 1:** Imaging protocol for patients.

Parameters	Axial T1WI	Axial T2WI	Sagittal T2WI	Coronal T2WI	Axial DWI	Sagittal DCE
TR (ms)	160	1750	4050	6240	6900	160
TE (ms)	10	80	120	93	80	10
Slice thickness (mm)	5	5	5	5	5	5
Gap (mm)	1	1	1	1	1	1
FOV (mm)	350 × 275	350 × 275	280 × 245	350 × 275	350 × 275	280 × 245
Matrix	256 × 245	256 × 245	375 × 275	375 × 275	128 × 128	375 × 275

*TR* repetition time, *TE* echo time, *FOV* Field of view, *DCE* dynamic contrast enhanced.

### Image analysis

Two experienced radiologists, Gu and Zhang (with 8 and 10 years of experience in gynecologic imaging, respectively) independently reviewed the MRI images. Discrepancies between the results were resolved through discussion.

#### Quantitative measurements

Tumor size, ADC value, relative T1 value (r-T1), and relative T2 value (r-T2) were measured. Regions of interest (ROIs) were drawn on the solid part of the tumor as large as possible to cover the entire area. During the measurements, cystic and hemorrhagic areas were avoided. The r-T1 and r-T2 values were calculated by dividing the signal intensity by that of the iliopsoas muscle on T1WI and T2WI, respectively.

#### Qualitative measurements

The following qualitative MRI features were recorded: tumor margin (well-defined or ill-defined); tumor component (solid, solid-cystic, cystic); shape (smooth or lobulated); signal intensity on DWI (hyperintensity or isointensity compared with the adjacent myometrium); tumor enhancement (mild, moderate, and avid enhanced); associated myoma (present or absent); stone paving sign (present or absent); ovary sign (present or absent); and pelvic-free ascites (present or absent). The tumor component was defined as solid (80–100% solid component), cystic (80–100% cystic component), or solid-cystic (others). The tumor enhancement type was graded as follows: (1) mild enhancement (less than the myometrium), (2) moderate enhancement (similar to the myometrium), or (3) avid enhancement (more than the myometrium). The stone paving sign was defined as a non-specific sign of tumor characterized by diffuse pebble-like hypo-intensity associated with thickened septa and mimicking a paving stone. An ovarian sign was defined as the presence of an ipsilateral ovary on any of the MR images.

### Statistical analysis

Statistical analysis was performed using IBM SPSS (version 22.0; IBM Corp., NY, USA) and R software (v. 4.0.4; http://www.r-project.org/). The inter-observer agreement between the two readers was compared using the kappa test. Univariate analysis was conducted to compare clinical and imaging features between the groups. Data normality was evaluated using the Shapiro–Wilk test for quantitative variables. Continuous data were compared using the Student’s t-test or Mann–Whitney U-test. Categorical variables were compared using the chi-squared test or Fisher’s exact test. Multivariable logistic regression analyses were used to identify factors that could independently differentiate OFs from BLMs. Three differentiation models were built based on the quantitative and qualitative variables. Specifically, Model 1 was based on preoperative quantitative variables (age, tumor size, ADC value, r-T, and r-T2). Model 2 was established based on qualitative features (menopausal status, tumor margin, component, shape, DWI signal, tumor enhancement, associated myoma, stone paving sign, ovary sign, and ascites). Model 3 was constructed from a combination of all quantitative and qualitative variables that were found to be significantly different between the two groups. Given the small sample size, fivefold cross-validation method^9^ was used for internal validation to avoid overfitting models and improve accuracy. Logistic regression models were established using fivefold cross-validation, and the area under the ROC curve (AUC) was calculated. Based on the AUC, the optimal training and validation sets were selected, and a logistic regression model was established. The optimal accuracy, sensitivity, specificity, positive predictive value, and negative predictive value of each model were determined based on the threshold that maximized Youden index of the cross-validation results. Subsequently, a nomogram was established to visualize the model based on the results of multivariable logistic analysis. The Hosmer–Lemeshow test was performed to assess the calibration capability of the nomogram. Decision curve analysis^10^ (DCA) was performed to assess the clinical usefulness of the nomogram by calculating net benefits. Statistical significance was set at *P* < 0.05.

## Results

### Clinical characteristics

**Figure 1:**
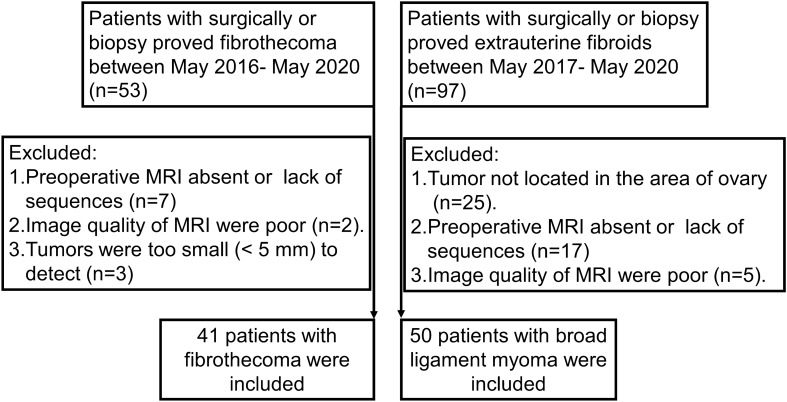
The flow chart of patient selection.

A total of 91 patients were included in the final analysis after excluding 69 patients who met the exclusion criteria (Fig. 1). Among them, 41 patients were assigned to the OF group (mean age, 52.85 ± 16.33 years old), while 50 patients were assigned to the BLM group (mean age, 46.4 ± 9.27 years old). Most patients in the OF group were postmenopausal (*n* = 29, 70.7%). A significant difference was found in age and menopausal status between the two groups (OF, *P* = 0.02; BLM, *P* = 0.002). According to histopathological findings, 25 patients were classified as having fibromas, 13 as having fibrothecomas, and 3 as having thecomas. Pathological characteristics are shown in Tables 2 and 3.

**Table 2:** Histopathological results of 91 lesions.

Pathological diagnosis	Lesion number	Percentage (%)
OF	41	45.1%
Fibroma	25	27.5%
Fibrothecoma	13	14.3%
Thecoma	3	3.3%
BLM	50	54.9%

*OF* ovary fibrothecoma, *BLM* broad ligament myoma.

**Table 3:** Clinical and imaging features of Fibrothecoma and BLM.

Characteristics	OF (*n* = 41)	BLM (*n* = 50)	*P*-value
Age	52.85 ± 16.33	46.4 ± 9.27	0.02
Tumor size	8.35 ± 4.67	7.58 ± 3.85	0.62
ADC (× 10–3 mm2/s)	0.98 ± 0.21	0.94 ± 0.23	0.49
r-T1	1.05 ± 0.18	0.82 ± 0.17	< 0.01
r-T2	2.42 ± 1.10	1.59 ± 1.04	< 0.01
**Menopause status**			0.002
Premenopausal	12 (29.3%)	31 (62%)	
Postmenopausal	29 (70.7%)	19 (38%)	
**Tumor margin**			0.13
Well-defined	27 (65.9%)	40 (80%)	
Ill-defined	14 (34.1%)	10 (20%)	
**Component**			< 0.01
Solid	23 (56.1%)	48 (96%)	
Solid-cystic	7 (17.1%)	2 (4%)	
Cystic	11 (26.8%)	0 (0%)	
**Shape**			0.87
Smooth	22 (53.7%)	26 (52%)	
Lobulated	19 (46.3%)	24 (48%)	
**Signal on DWI**			0.93
Hyperintensity	25 (29.3%)	30 (60%)	
Isointensity	16 (29.3%)	20 (40%)	
**Enhancement**			< 0.01
Mild	19 (46.3%)	5 (10%)	
Moderate	16 (39.1%)	6 (12%)	
Avid	6 (14.6%)	39 (78%)	
**Associated myoma**			0.06
Present	9 (21.9%)	20 (40%)	
Absent	32 (78.1%)	29 (58%)	
**Stone paving sign**			< 0.01
Present	11 (26.8%)	33 (66%)	
Absent	30 (73.2%)	17 (34%)	
**Ovary sign**			< 0.01
Present	12 (29.3%)	33 (66%)	
Absent	29 (70.7%)	17 (34%)	
**Ascites**			< 0.01
Present	35 (85.4%)	21 (42%)	
Absent	6 (14.6%)	29 (58%)	

Values are given as *n* (%), mean ± SD (range).

*OF* ovary fibrothecoma, *BLM* broad ligament myoma, *ADC* apparent diffusion coefficient, *r-T1* relative T1 value, *r-T2* relative T2 value.

### MRI characteristics

The interobserver agreement was excellent (0.8–1) for all quantitative and qualitative measurements. The MRI findings of the OF and BLM groups are listed in Table 3. There were no significant differences between the two groups in terms of tumor size, ADC value, tumor margin, shape, or DWI signal. In contrast, the tumors in the OF group had higher r-T1 and r-T2 values in quantitative features than those in the BLM group. In the qualitative measurements, mild-to-moderate enhancement and cystic components were more frequently seen in the OF group than in the BLM group (all *p* < 0.01). Mild enhancement and moderate enhancement were found in 19 (46.3%) and 16 (39.1%) patients with OF, respectively, whereas avid enhancement was found in 39 (78%) patients with BLMs.

Regarding the tumor component, BLMs mostly appeared as solid component (96%), whereas OFs showed a partial cystic component (43.9%). Notably, there was a higher tendency for stone paving sign and ovary sign in tumors of the BLM group than in those of the OF group. Pelvic free ascites was commonly seen in OF patients (85.4%), whereas only 21 (42%) cases were observed in BLMs.

### Performance of the models

Multivariable logistic regression analyses were used to construct models to discriminate OF patients from BLM patients (Table 4). Among the quantitative variables in Model 1, age (OR = 0.950; 95% CI, 0.909–0.992), r-T1 (OR = 0.004; 95% CI, 0.001–0.014), and r-T2 (OR = 0.414; 95% CI, 0.225–0.761) were factors that could independently differentiate OF from BLM. The qualitative variables of enhancement (OR = 6.755; 95% CI, 2.477–18.418), stone paving sign (OR = 22.405; 95% CI, 3.359–149.46), ovary sign (OR = 4.787; 95% CI, 0.937–24.445), and pelvic-free ascites (OR = 0.091; 95% CI, 0.014–0.586) could independently differentiate OF from BLM in Model 2. The following factors were used to construct Model 3: r-T1 (OR = 0.002; 95% CI, 0.001–0.168), enhancement (OR = 4.762; 95% CI, 1.696–13.369), stone paving sign (OR = 19.475; 95% CI, 2.700–140.47), and ascites (OR = 0.051; 95% CI, 0.006–0.431).

**Table 4:** Multivariate logistic regression of three models for differentiation of fibrotecoma and BLM.

Model	Variables	Weight	OR (95% CI)	*P*-value
Model 1	Age	− 0.051	0.950 (0.909–0.992)	0.021
	r-T1	− 7.952	0.004 (0.001–0.014)	< 0.001
	r-T2	− 0.882	0.414 (0.225–0.761)	0.005
Model 2	Enhancement	1.901	6.755 (2.477–18.418)	< 0.001
	Stone paving sign	3.109	22.405 (3.359–149.46)	0.001
	Ovary sign	1.566	4.787 (0.937–24.445)	0.043
	Ascites	− 2.394	0.091 (0.014–0.586)	0.012
Model 3	r-T1	− 6.246	0.002 (0.001–0.168)	0.006
	Enhancement	1.561	4.762 (1.696–13.369)	0.003
	Stone paving	2.969	19.475 (2.700–140.47)	0.003
	Ascites	− 2.973	0.051 (0.006–0.431)	0.006

*OR* odds ratio, *AUC* Area under curve, *SEN* sensitivity, *SPC* specificity, *r-T1* relative T1 value, *r-T2* relative T2 value.

**Figure 2:**
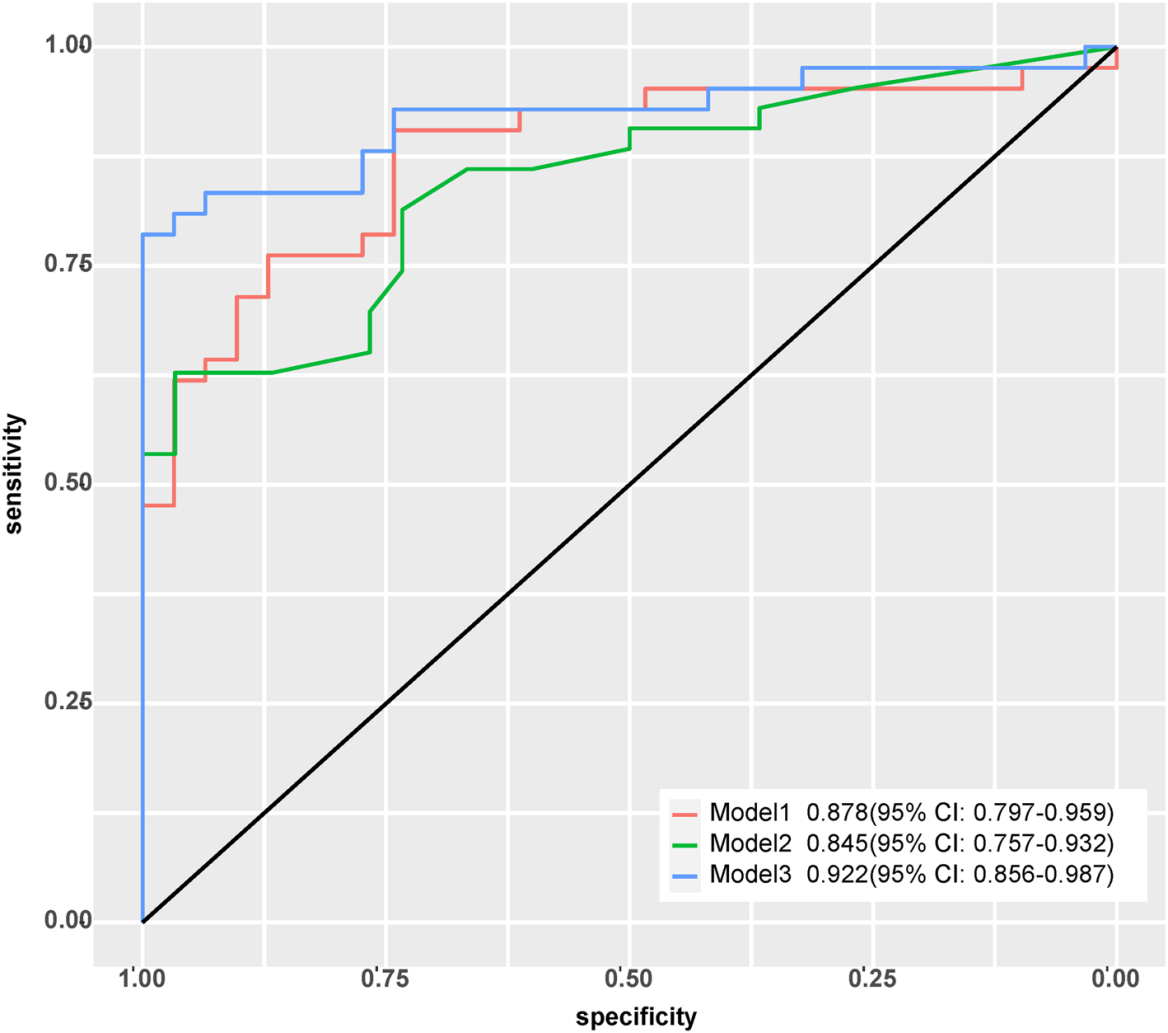
ROC curves of three models.

The AUC of Model 1, 2 and 3 was 0.88 (95% CI: 0.79–0.96), 0.85 (0.76–0.93), and 0.92 (0.86–0.98), respectively. The cutoff value, accuracy, sensitivity, specificity, positive predictive value, and negative predictive value of the three models are shown in Table 5. The ROC curves of the models are shown in Fig. 2.

**Table 5:** The performance of three diagnostic models.

Model	AUC (95% CI)	SEN	SPE	PPV	NPV	ACC	Cut-off value
Model 1	0.88 (0.79–0.96)	72%	90.2%	90%	72.5%	80.2%	0.21
Model 2	0.85 (0.76–0.93)	66%	78%	78.6%	65.3%	71.4%	0.69
Model 3	0.92 (0.86–0.98)	76%	95.1%	95%	76.4%	84.6%	0.38

Abbreviations: *SEN* sensitivity, *SPE* specificity, *PPV* positive predictive value, *NPV* negative predictive value, *ACC* accuracy.

**Figure 3:**
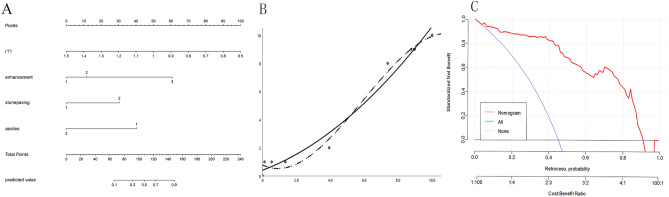
(**A**) The nomogram is based on Model 3, incorporating the quantitative and qualitative MRI signature. When using the nomogram, specific factors were located according to its measurements, and then a line straight up to the points axis was drawn to get the factor’s score. A total of points was obtained by summing the scores of all the involved factors. Finally, the probability of BLM was determined by drawing a line straight down to the bottom axis. (**B**) Calibration curves for the nomogram. The y-axis represents BLM’s actual rate in the patients; the x-axis represents the nomogram-predicted probability of BLM. Hosmer–Lemeshow test showed that the nomogram's predicted efficiency is in good agreement with the actual condition (X2 = 6.855, *p* = 0.552 > 0.05). (**C**) Decision curve analysis (DCA) showed that the nomogram had a high net benefit in a wide range of threshold levels.

**Figure 4:**
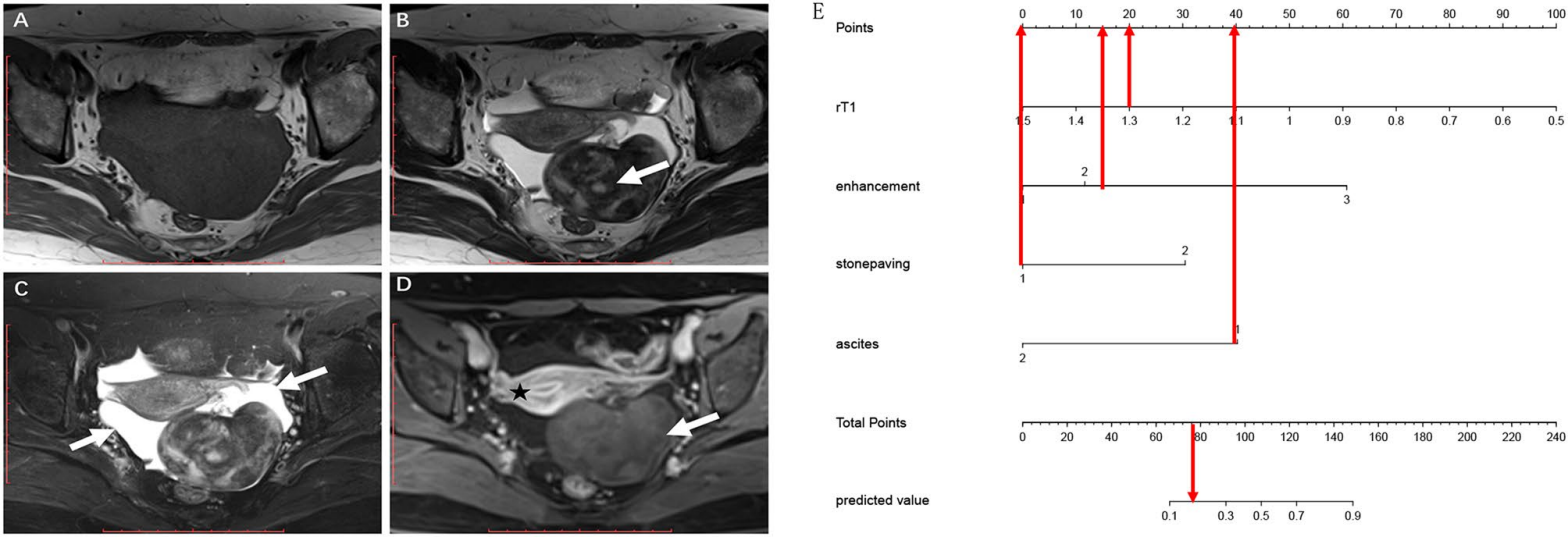
A 65-year-old woman with an OF. (**A**) Axial T1-weighted imaging shows a hypointense tumor in the left ovary. (**B**) On T2-weighted imaging, the tumor exhibits the cystic part (white arrow). (**C**) On the fat-saturated T2WI, the free ascites (white arrow) were seen in the pelvis. (**D**) The tumor showed mild contrast enhancement (white arrow) comparing with the uterus (asterisk). (**E**) This tumor’s total score is 75 (r-T1 = 23, enhancement = 12, stone paving = 0, ascites = 40) with a predicted value of 0.15 calculated by the nomogram, the corresponding probability of OF is 85%.

**Figure 5:**
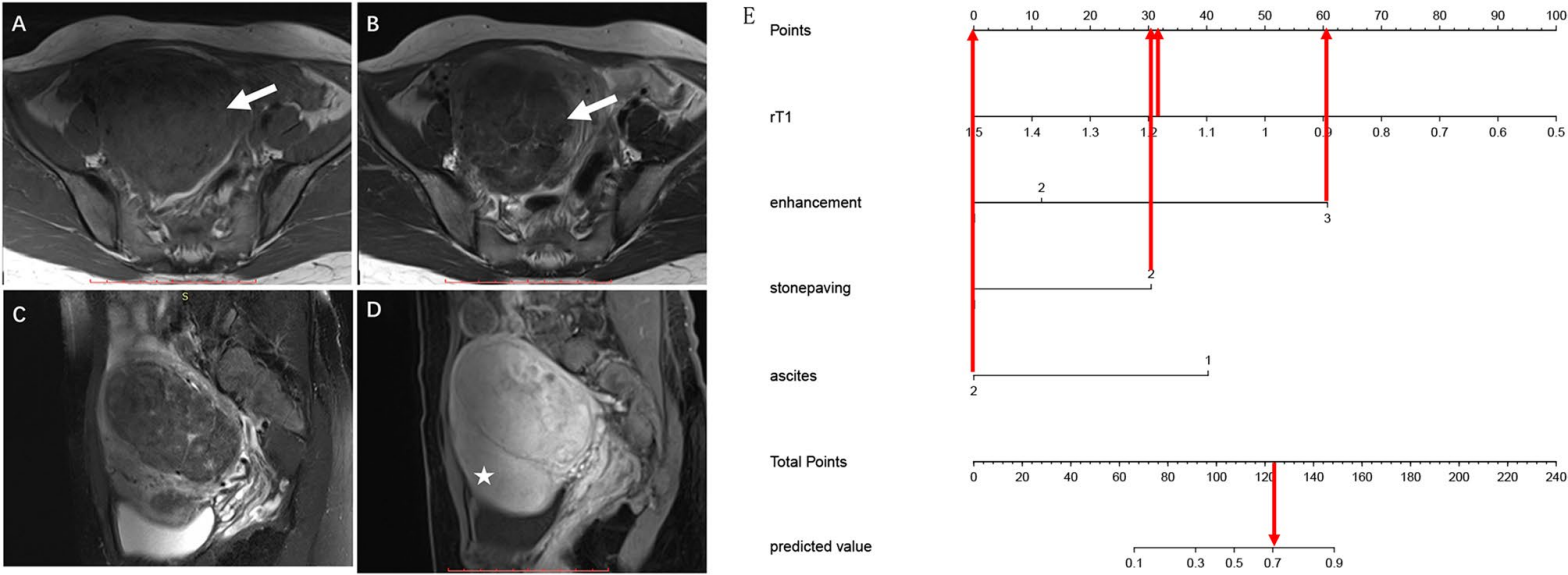
A 43-year-old woman with a BLM. (**A**) Axial T1-weighted imaging shows a hypointense tumor in the right adnexal region. (**B**) On T2-weighted imaging, the tumor exhibits a stone paving sign (white arrow). (**C**) None pelvic effusion was found in the pelvic. (**D**) The tumor shows avid contrast enhancement (white arrow) comparing with the uterus (asterisk). (**E**) This tumor’s total score is 124 (r-T1 = 32, enhancement = 61, stone paving = 31, ascites = 0) with a predicted value of 0.71 calculated by the nomogram; the corresponding probability of BLM is 71%.

A nomogram was constructed based on the multivariate logistic regression results of Model 3 (Fig. 3A). The calibration curve (Fig. 3B) of the nomogram showed that the calibration between the predicted and actual outcomes of tumor identification was good. DCA analysis showed that the nomogram was clinically useful, with a high net benefit over a wide range of threshold levels (Fig. 3C). Figure 4 shows a representative case of the OF group, whereas Fig. 5 shows a representative case of the BLM group.

## Discussion

In this study, we developed an MRI-based nomogram for the preoperative differentiation of OFs from BLMs. This nomogram successfully differentiated OF from BLM based on preoperative MRI features, with good discrimination efficiency.

In the present study, Model 1, based on preoperative quantitative variables, showed the potential to preoperatively differentiate OF from BLM, with an AUC of 0.88, and an ACC of 80.2%. Both the AUC and ACC of Model 2, based on qualitative MRI features, were lower than those of Model 1 were. Model 3 showed the best performance of the three models with an AUC of 0.92 and ACC of 84.6%, indicating that MRI features can preoperatively differentiate OF from BLM. The results of Model 3 were used to construct a nomogram for the preoperative differentiation of OF from BLM. In the present study, age and r-T1 and r-T2 values were identified as valuable quantitative factors that could differentiate OF from BLM. Patients with OF were significantly older than those with BLM were. As a uterine fibroid, BLM usually affects women of reproductive age^11^. However, most patients with OF are in their fifth and sixth decades of life^12–15^. The mean age of patients in the present study was 52.85 years for OF and 46.40 years for BLM. Most women with OF were postmenopausal (70.7%), while only 38% were postmenopausal in the BLM group. Patients in the BLM group showed significantly lower r-T1 and r-T2 values than those in the OF group (*p* < 0.05). Previous studies have qualitatively compared the signal characteristics of OF or BLM with adjacent myometrium^16–19^. OF and BLM are all fibrous-containing tumors; hence, they are mostly isointense to hypointense on T1WI and T2WI compared to the adjacent myometrium (2, 5, 8, 14).

No significant difference in signal intensity was observed in most previous studies^5,20^. In the present study, T1WI and T2WI signal intensities were quantitatively and objectively evaluated. We found that r-T1 and r-T2 were significantly different between the two groups. Model 1, which included age, r-T1, and r-T2, showed good performance in differentiating between the two tumors. The study findings suggest that the patients' quantitative features play a significant role in differentiating OFs from BLMs.

Moreover, we evaluated qualitative parameters for differential diagnosis. Menopause status, tumor component, enhancement pattern, stone-paving sign, ovary sign, and ascites were significantly different between the OF and BLM groups. Multivariate logistic regression analysis showed that paving signs, ovarian signs, and ascites could differentiate between the two tumors. Further analysis revealed that the enhancement of the OF was significantly lower than that of the BLM. Thomassin-Naggara et al.^5^ evaluated the ability of dynamic contrast-enhanced (DCE) MRI to distinguish between OF and subserous uterine leiomyomas. They found that the enhancement measurements were lower for OF than for uterine leiomyomas with respect to the maximal enhancement or enhancement rate. This feature reflects the differential blood supply between the two tumors. The vasculature of uterine leiomyomas is characterized by sizable branches of the uterine artery and dilated capsular vessels. In contrast, OFs tend to have very few or no arterial supply. In the present study, 14.6% of the OFs showed avid enhancement and were proven to be thecomas and fibrothecomas with a sizeable number of theca cells. These features indicated that it was more likely to be a fibrothecoma with more theca cell components or thecoma when an ovarian lesion showed marked enhancement. Moreover, the stone-paving sign was an independent predictor of BLM. This imaging manifestation is in line with the morphology of the gross specimen and arrangement of smooth muscle cells.

Notably, OF contained a cystic portion or stromal edema, whereas most BLM tumors contained a pure solid mass. We presume that this discrepancy explains the difference in stone-paving sign. Furthermore, the ipsilateral ovary was present in 66% of the BLM cases and in only 26.8% of the patients. Several studies have evaluated ovarian signs in patients with OF^12,15,19^. Ovarian signs were identified in 86% and 46% of patients by^12,15^, indicating that the female pelvic mass did not originate from the ipsilateral ovary. Our results showed that the detection rate of ovarian signs was lower in the OF group than in the BLM group. This implies that the ovaries were obscured in patients with masses of ovary-origin. We speculate that some exogenous growth patterns of OFs also contribute to the typical appearance of the ipsilateral ovaries. Many OFs with peritoneal and pleural effusions are considered Meigs syndrome, which mimics malignant pelvic masses^21^. As reported by Iyer et al.^22^, ascites disappear after removal of the primary tumor. Although the syndrome is present in a small proportion of ovarian tumors, pelvic effusion is commonly observed in benign or malignant gynecological tumors. However, pelvic fluid is rarely observed in uterine leiomyomas^23,24^, indicating that ascites is also an important sign in the differential diagnosis.

As previously mentioned, MRI features are useful for preoperative differentiation between OF and BLM. In this context, we assessed the predictive efficacy of Model 3 by combining all the qualitative and quantitative features. The results revealed that Model 3 had the highest AUC and ACC among the three models. These outcomes indicate that the combined model could correctly differentiate OF from BLM. Owing to its noninvasive nature, the preoperative performance of this model is clinically acceptable and meaningful for patients in helping to make clinical decisions. Based on Model 3, a nomogram was established for clinical application. Furthermore, the nomogram is a practical tool for describing the value of an individual factor in the scoring system. To the best of our knowledge, the present study is the first to establish a nomogram to differentiate OF from BLM preoperatively.

Study limitations: First, only imaging features were used to construct the model, without including a single laboratory feature. Further studies, including serum biomarkers, should be performed to explore the effects of laboratory tests. Second, the sample size enrolled in this study was small; hence, a prospective study with a larger sample size is required. Third, the performance of the constructed model was not clinically verified. Further studies with more samples and validation cohorts should be performed to validate our results. Fourth, the predictive model obtained in this study was not externally verified, and external data should be collected for external validation.

## Conclusion

In summary, preoperative MRI parameters can be used to differentiate OFs from BLMs. The nomogram, based on Model 3, showed good discriminatory ability between OF and BLM.

## Data Availability

The data that support the findings of this study are available on request from the corresponding author.
